# The Risk of Subsequent Deep Vein Thrombosis and Pulmonary Embolism in Patients with Nontyphoidal Salmonellosis: A Nationwide Cohort Study

**DOI:** 10.3390/ijerph17103567

**Published:** 2020-05-19

**Authors:** Renin Chang, Den-Ko Wu, James Cheng-Chung Wei, Hei-Tung Yip, Yao-Min Hung, Chih-Hsin Hung

**Affiliations:** 1 Institute of Biotechnology and Chemical Engineering, I-Shou University, Kaohsiung 840, Taiwan; rhapsody1881@gmail.com (R.C.); dkw3275@gmail.com (D.-K.W.); 2 Department of Emergency Medicine, Kaohsiung Veterans General Hospital, Kaohsiung 813, Taiwan; 3 Department of Recreation Sports Management, Tajen University, Pingtung 90741, Taiwan; 4 Division of Allergy, Immunology and Rheumatology, Chung Shan Medical University Hospital, Taichung 402, Taiwan; wei3228@gmail.com; 5 Institute of Medicine, Chung Shan Medical University, Taichung 402, Taiwan; 6 Graduate Institute of Integrated Medicine, China Medical University, Taichung 404, Taiwan; 7 Management Office for Health Data, China Medical University Hospital, Taichung 404, Taiwan; fionyip0i0@gmail.com; 8 Department of Internal Medicine, Kaohsiung Municipal United Hospital, Kaohsiung 804, Taiwan; 9 School of Medicine, National Yang Ming University, Taipei 112, Taiwan; 10 Yuh-Ing Junior College of Health Care and Management, Kaohsiung 807, Taiwan

**Keywords:** nontyphoidal salmonellosis, venous thromboembolism, deep vein thrombosis, pulmonary embolism, cohort study

## Abstract

The purpose of this study was to evaluate the deep vein thrombosis (DVT) and pulmonary embolism (PE) risk among patients with a diagnosis of nontyphoidal salmonellosis (NTS) in an Asian population. The risk was analyzed in a cohort of 17,855 patients newly diagnosed with NTS and 71,420 individuals without NTS using a hospitalization claim dataset. Both groups were matched by age, sex, and index date as an original analysis. A Cox proportional-hazards regression model was applied to estimate the risk of DVT and PE, accounting for any competing event (death). With a follow-up of 4.94 (±3.93) years in the NTS group and 6.30 (±3.67) years in the non-NTS group, the adjusted subhazard ratios (SHRs) of DVT and PE were 1.83 (95% CI 1.44–2.31) and 1.84 (95% CI 1.30–2.60). The NTS group had an increased risk of DVT and PE compared with the control group in all of the age subgroups. Stratified analyses showed that patients aged 18–39 years in the NTS group had significantly higher DVT and PE risks compared with patients of the same age in the non-NTS group (aHR, 5.95; 95% CI, 2.22–15.91 for DVT; aHR 6.72; 95% CI, 2.23–20.30 for PE). The *P*-value for interaction between age and exposure of NTS is <0.001 for DVT and 0.004 for PE in our sub-group analyses. The findings were cross-validated by a re-analysis with propensity score matching (PSM), and that revealed consistent results. Despite of low absolute risk, clinicians should be aware that patients with an NTS hospitalization history is at increased risk for VTE especially when assessing patients coincident with other VTE risk factors.

## 1. Introduction

Salmonellae is one of the leading global causes of diarrheal diseases and responsible for a substantial burden of food-borne illnesses. Salmonellae, able to survive weeks in a dry environment, and months in water, are motile Gram-negative intracellular bacteria with bewildering array of clinical characteristics, from uncomplicated gastroenteritis to focal metastatic infections. Nontyphoidal salmonellosis (NTS) is characterized by acute onset of fever, abdominal pain, and diarrhea. Symptoms can be severe in the young, the elderly, and patients with multiple co-morbidities and weakened immunity. Venous thromboembolism (VTE) is a collective term that includes deep vein thrombosis (DVT) and pulmonary embolism (PE), which can be fatal [1]. PE is one of the foremost causes of cardiovascular mortality, overtaken only by stroke and acute myocardial infarction [2]. The interplay between Salmonellae and the development of thrombosis has been known for more than a century, and subsequent studies have confirmed that acute infections are a risk factor for subsequent VTE [3,4,5,6,7,8]. Several animal models have shown the impact of bacterial infections on subsequent inflammation and coagulation in vivo. In contrast to other pathogens, such as *Staphyloccocus aureus*, *Klebsiella pneumoniae*, and *Pseudomonas aeruginosa*, where the hosts’ responses including increased platelet aggregation can last from minutes to hours, *Salmonella typhimurium* appears to initiate infection-driven thrombosis by an alternative pathway with increased platelet aggregation, for example in the liver vasculature, lasting for several weeks [9]. In another recent study using mouse bone marrow–derived macrophages infected with *Salmonella typhimurium*, it was demonstrated that, even when restricted in the macrophages, some NTS can secret effectors by the Salmonella pathogenicity island 2 type III secretion system to induce macrophage polarization from pro-inflammatory innate immune responses to anti-inflammation and thus undermine the host’s immune defense and survive for extended periods [10]. Based on these studies of pathogen-induced thrombosis and bacterial persistence in animal models, we hypothesized that patients with NTS have an increased risk of VTE for a prolonged period following their acute infection. However, there is little epidemiologic evidence to support this hypothesis. Thus, we performed a nationwide population-based cohort study to analyze the risk of VTE in people with a history of NTS.

## 2. Materials and Methods 

### 2.1. Data Source and Study Design 

This study was approved by the Research Ethics Committee of China Medical University and Hospital in Taiwan (CMUH-104-REC2-115). Because the study applied de-identified the secondary dataset, released for research purposes, the need for informed consent was waived. 

NHIRD contains health care data of more than 99% of the 23.6 million residents in Taiwan since 1995. All medical claims were sent to the Bureau of National Health Insurance (NHI) for validation and reimbursement. The registration files included demographics, all types of medical visits, and prescriptions codes; before 2016, the procedure and diagnostic codes were based on the International Classification of Diseases, 9th Revision, Clinical Modification (ICD-9-CM). To ensure the accuracy of disease diagnosis, the Bureau of NHI randomly reviewed the medical charts of one in 20 inpatient claims. Based on the original claim data in Taiwanese National Health Insurance Research Database (NHIRD), several specific data subsets are constructed for research purposes. We conducted a population-based cohort study by retrieving the inpatient dataset, a subset from NHIRD, including the original claim data of inpatients by admission. 

### 2.2. Study Population

All adult patients aged 20 years or older with a first-time discharge diagnosis of NTS in the period from January 2000 to December 2013 were identified within the inpatient dataset. NTS diagnosis was defined as a discharge diagnosis using the ICD-9-CM code 003.XX. Administrative coding for NTS has been applied in a previous study [11]. The index date was defined as the NTS diagnosis date. Patients were excluded if they were younger than 20 years of age, had a history of NTS or VTE before their entry into the study, or died during hospitalization for NTS. To avoid selection bias, the control group (no history of NTS) was randomly selected by computer from the database. The control individuals were matched according to sex, age, and index dates. Index dates for subjects in the control group were randomly assigned and corresponded to individuals in the NTS group. Both groups fit the same inclusion and exclusion criteria. In the primary analysis, both cohorts were individually matched at a ratio of 1:4 by sex, gender, and index date. We re-analyzed the data by propensity score matching to test the robustness of the study findings.

### 2.3. Outcome and Relevant Variables 

The main outcome was the diagnosis of VTE during the follow-up period. All patients were followed until the occurrence of VTE, until withdrawal from health insurance, or until 31 December, 2013. To mitigate the effects of potential confounders, we included the following covariates on the dataset for analysis: age, gender, and relevant medical co-morbidities for the development of VTE, including diabetes mellitus (ICD-9-CM code 250), hypertension (ICD-9-CM codes 401-405), hyperlipidemia (ICD-9-CM code 272), chronic obstructive pulmonary disease (ICD-9-CM codes 491, 492, 496), asthma (ICD-9-CM code 493), coronary artery disease (ICD-9-CM codes 410-414), stroke (ICD-9-CM codes 430-438), chronic kidney disease (ICD-9-CM code 585), cancer (ICD-9-CM codes 140-208), sleep apnea (ICD-9-CM codes 327.23, 780.51, 780.53, 780.57), rheumatoid arthritis (ICD-9-CM code 714), atrial fibrillation (ICD-9-CM code 427.31), chronic liver diseases (ICD-9-CM code 571.4), pregnancy (ICD-9-CM codes 640.x1–676.x1, 640.x2–676.x2, 650–659 and with procedure codes 72–74), and lower limb fracture or surgery (ICD-9-CM codes 820–823 and with procedure codes 81.51, 81.52, 81.53, and 81.54). Information was obtained by tracing all the inpatient records in the NHI database within 2 years before the index date.

### 2.4. Statistical Analysis 

For analysis of general characteristics of the individuals in the both cohorts, categorical and continuous variables were analyzed using chi-square and Student t-tests, respectively. We defined person-years by calculating the sum of the follow-up period for each individual. The individual follow-up period was obtained from the index date to the occurrence of VTE, withdrawal from the NHI program (for examples, people missing over 6 months, emigration, in prison, or death), or the end of 2013—whichever occurred first. The incidence rate was calculated according to the number of occurrences and person-years. To assess the risk of developing subsequent VTE, we performed Cox proportional hazards regression analysis to obtain crude and adjusted hazards ratios (HRs) and 95% confidence intervals (CIs) between the two cohorts. Cox’s regression models were adjusted by age, gender, and all co-morbidities. The Kaplan–Meier method was used to describe the cumulative incidence of VTE among the two groups; differences between the two groups were evaluated using the log rank test. All data analyzed were performed with SAS® (version 9.4; SAS Institute, Inc., Cary, NC, USA). Statistical significance was set at a *p*-value < 0.05. We applied the Aalen Johansen estimator for analysis of the competing risk of death. 

## 3. Results

### 3.1. Patient Characteristics

We included 89,275 participants in this study (NTS group: 17,855; non-NTS group: 71,420). Table 1 reveals that, in the primary analysis, the incidences of co-morbidities were higher in the NTS group than the control group, and we applied regression analysis to adjust for the covariate differences between the two groups. We re-analyzed the data by propensity score matching (PSM) and have provided information on the patients’ demographic characteristics, co-morbidities and mortality in the Supplementary Materials (Table S1).

### 3.2. Risk of DVT and PE

Table 2 presents the incidence rates and hazard ratios (aHRs) of DVT and PE in the primary analysis (i.e., before PSM). The incidence rate of DVT in the NTS group (12.36 per 10,000 person-years) was higher than among the non-NTS participants (4.77 per 10,000 person-years). PE was more prevalent in the NTS group (6.01 per 10,000 person-years) than in the non-NTS group (2.42 per 10,000 person-years). The NTS group had a 2.35-fold higher risk of DVT (95% CI = 1.86–2.98) and PE by 2.36 (95% CI = 1.69–3.30). We used the Aalen Johansen estimator for the analysis of a competing risk of death. The sub-hazard ratios (SHRs) of DVT and PE were 1.83 (95% CI = 1.44–2.31) and 1.84 (95% CI = 1.30–2.60), respectively. Compared with subjects aged less than 40 years (the reference age group), those aged over 65 years had the highest risk of developing DVT (aHR = 5.58; 95% CI, 3.38–9.22) and PE (aHR = 4.51; 95% CI, 2.46–8.26). 

### 3.3. Analysis Stratified by Sex, Age, and Co-Morbidities

Table 3 demonstrates the associations of risks of VTE by subgroup analyses in terms of sex, age, and co-morbidities. Sex-subgroup analysis revealed that compared with women without NTS, women with NTS had a 2.34-fold higher risk of DVT (95% CI 1.67–3.28); men with NTS had a 2.26-fold higher risk of DVT than men without NTS (95% CI 1.63–3.14). Compared with men without NTS, men with NTS had a significantly higher risk of PE (aHR = 3.04, 95% CI 1.94–4.75). In the age subgroup analysis, patients with NTS had an increased association with the risk of DVT and PE in people aged over 65 (for DVT, aHR = 1.68, 95% CI 1.23–2.30; for PE, aHR = 1.54, 95% CI 0.96–2.47), and patients with NTS had the highest risk of developing DVT and PE in those aged below 40 (for DVT, aHR = 5.95, 95% CI 2.22–15.91; for PE, aHR = 6.72, 95% CI 2.23–20.28) compared with matched non-NTS age subgroups. The *P*-value for interaction between age and exposure of NTS is <0.001 for DVT and 0.004 for PE in age subgroup analyses. In the co-morbidities subgroup analysis, in people without mentioned co-morbidities, having a new diagnosis of NTS entailed a significantly higher risk of developing PE than those without NTS. The *p*-value for interaction between co-morbidities and exposure of NTS is <0.001 for PE.

### 3.4. Sensitivity Analysis

Table 4 shows that the results were consistent in the two different regression models. We re-analyzed the study by performing PSM to minimize the confounding effects of the mentioned comorbidities on the incidence of VTE. After PSM at a 1:4 ratio, accounting for a competing risk of death, Table S1 shows the baseline comorbidities in both groups, and Table 4 shows that the SHRs of DVT and PE were 1.69 (95% CI = 1.34–2.13) and 1.71 (95% CI = 1.21–2.40), respectively. 

### 3.5. Time Trends for Risk of DVT and PE

The incidence rate of DVT and PE in the NTS group demonstrated a time-dependent trend during the follow-up period (Table 3). The risk of DVT in the NTS group, relative to the non-NTS group, was highest after the first 12 months (7.34 per 10,000 years; aHR = 1.75, 95% CI 1.31–2.34); the risk of PE was highest between 3 and 12 months (11.1 per 10,000 years; aHR = 1.56, 95% CI 1.03–2.38). 

### 3.6. Cumulative Incidences Completing Risk Analysis of DVT and PE in NTS and non-NTS Groups

**Figure 1:**
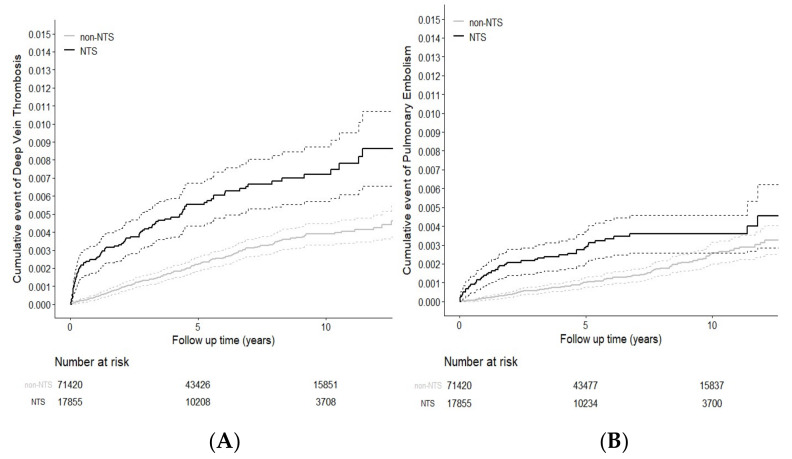
Cumulative incidence competing risk (death) for deep vein thrombosis (DVT) (**A**) and pulmonary embolism (PE) (**B**) among patients with and without NTS.

The Kaplan–Meier graphs (Figure 1(A) and 1(B)) illustrated that the cumulative incidences with death as competing risk for DVT and PE were higher in the NTS group than in the comparison group (log-rank test *p* < 0.001).

## 4. Discussion

This is the first nationwide population-based cohort study to provide epidemiological evidence indicating people with NTS are associated with a higher risk of new-onset DVT (1.83-fold) and PE (1.84-fold) in the Asian population. Patients with a diagnosis of NTS had a significantly higher proportion of comorbidities in comparison with subjects in the non-NTS group. A diagnosis of NTS remained an independent risk factor for subsequent VTE after adjusting for sex, age and co-morbidities. We conducted a re-analysis of our data using PSM that validated these findings. It is notable that males with NTS had a higher risk for PE than females. The study enrolled NTS inpatients as the study population for better disease coding validity. It should be noted, however that as an observation retrospective cohort study, individuals with NTS who never sought medical aid and/or had asymptomatic NTS or mild gastroenteritis were not included in this study. Patients with a discharge diagnosis of NTS have a higher risk of developing DVT and PE in the months following their discharge.

Animal models have shown that bacterial infections—such as *Staphylococcus aureus*, *Klebsiella pneumoniae*, and *Pseudomonas aeruginosa*—are associated with subsequent inflammation and coagulation, and increased platelet aggregation which can last from minutes to hours [12]. A contrast between the Salmonella model of infection-related thrombosis and these other bacterial models is the timing of thrombosis formation–Salmonellae-related thrombosis develops much later. Previous studies indicate that patients with acute infections have an increased risk of VTE [13], especially within the first 2 weeks [14]. Dalager-Pedersen et al. estimated the relationship between VTE and community-acquired bacteremia using a population-based cohort study, and they found the adjusted 90-day odds ratio for VTE to be 1.9 [15]. Mejer et al. indicated a high risk of VTE, as well as an overall hazard ratio from 15.6 within the first month, to 4.5 up to one year following an episode of *Staphylococcus aureus* bacteraemia [16]. However, epidemiologic evidence regarding NTS and thrombosis risk is lacking. This study determined that people with a history of hospitalization for NTS have a risk of subsequent DVT and PE for a longer period following discharge. The risk and timing of DVT and PE were further analyzed separately, indicating an increased DVT risk at more than 12 months after the index date, with the highest rate of PE occurring in the 3–12 months after the index date. However, we did not investigate the longer-term risk of VTE after NTS, as the study design did not include information about co-morbidities after study entry as time-varying covariates in the Cox models. 

The epidemiological causes of increased new-onset VTE in patients with a past history of NTS are unclear. Thromboses are life-threatening complications of systemic infections [17,18,19,20,21]. However, a multicenter prospective study showed that the incidence of VTE remained increased, despite the use of thrombo-prophylaxis in patients with sepsis [22], indicating that the prothrombotic risk is high. Recent studies have attempted to unravel the pathogenesis of NTS-related VTE. Firstly, it has been described that Salmonellae toxins enhance macrophage adhesion and translocation through endothelial cells, promoting phagocytosis of the bacteria into the macrophage—delivering the bacteria into the sub-endothelial space where it may remain in a dormant status for a long period [23]. A recent study has also shown that *Salmonella typhimurium* can adhere to and penetrate human microvascular endothelial cells by rearrangement of the actin cytoskeleton of host cells, which appears to be encoded by the Salmonella pathogenicity island 1 (SPI-1) locus [24]. Secondly, recent studies have shown that immune cells and the inflammatory process are prothrombotic [25]. Immune-mediated thrombosis has a role in human pathogen defenses; for example, immune cells and specific thrombosis-related molecules forms an intravascular scaffold for containment and destruction of pathogens [26]. Animal studies reveal that aberrant immune-mediated activation of thrombosis may be a key event in the initiation and propagation of DVT [27]. Investigators have described a mouse model of systemic *Salmonella typhimurium* infection that triggers thrombosis within blood vessels (persisting for weeks) that occurs through the ligation of C-type lectin-like receptor-2 on platelets to podoplanin exposed by damage to the vessel wall [9]. Although such *S. typhimurium*-mediated thrombosis is not altered by aspirin or clopidegrel [28], the inhibition of podoplanin-CLEC2 interactions with monoclonal antibodies does protect against DVT formation in this animal model [25]. Thirdly, neutrophils, the major host defense cells against microbial pathogens, have been recently described to be involved in a process termed “netosis.” When stimulated by certain bacteria, neutrophils release neutrophil extracellular traps (NETs) [29]—comprised of negatively charged neutrophil DNA backbones decorated with antimicrobial factors such as elastase, cathepsin G, and proteinase 3—that are capable of cleaving the virulence factors of *Shigella flexneri*, *Salmonella typhimurium*, and other pathogens [29,30,31]. *Salmonella typhimurium* has been shown to stimulate NETs that consequently trap and eliminate Salmonellae via granular proteins and H2A histone [30]. However, uncontrolled netosis may lead to extensive thromboses and is associated with subsequent ischemia [31,32]. Interestingly, several studies showed that neutrophils in older individuals generally “cast” fewer NETs [33,34]. Interestingly we observe that NTS has a higher association with subsequent VTE in younger patients, aged <40 years, who may have a stronger immune response (DVT, aHR = 5.95, 95% CI 2.22–15.91; PE, aHR = 6.72, 95% CI 2.23–20.28 when compared to control patients of the same age).

Several limitations of this study should be mentioned. First, NHIRD does not provide detailed demographics such as lifestyle information, tobacco consumption, body mass index, physical activity, or diet habits. Second, the hospitalization claim dataset from NHIRD does not include laboratory reports, indications of disease severity, and drug information, such as the use of anti-contraceptive drugs or antibiotics. Finally, a retrospective cohort study design is subject to biases due to unknown confounders. However, by analyzing these data using multivariable models and a sensitivity analysis, we have attempted to attenuate potential biases from unmeasured associated confounders.

## 5. Conclusions

In this population-based retrospective cohort study, NTS was associated with an increased risk of new-onset DVT and PE. Notably, the highest increase in relative risk of VTE occurs in younger patients (18–39 years) where the absolute risk of VTE is low. Nonetheless, clinicians should be aware that patients with a history of hospitalization with NTS are at increased risk for VTE within the first year following discharge, especially in situations where other VTE risk factors are present (e.g., post-operative, during prolonged immobility, pregnancy, etc.).

## Figures and Tables

**Table 1:** Baseline characteristics of the nontyphoidal salmonellosis (NTS) and non-NTS groups.

	Nontyphoidal Salmonellosis		*p* Value
	No (*N* = 71,420)	Yes (*N* = 17,855)	
	*n* (%)	*n* (%)	
Age, years			1.00
18–39	18,900 (26.5)	4725 (26.5)	
40–64	26,048 (36.5)	6512 (36.5)	
≥65	26,472 (37.1)	6618 (37.1)	
Mean (SD) *	55.3 (19.7)	55.4 (19.7)	0.62 *
Gender			1.00
Female	32,204 (0.45)	8051 (0.45)	
Male	39,216 (0.55)	9804 (0.55)	
Comorbidities	16,732 (23.4)	7951 (44.5)	<0.001
Hypertension	9376 (0.13)	4313 (0.24)	<0.001
Diabetes	4968 (0.07)	2854 (0.16)	<0.001
Hyperlipidemia	2219 (0.03)	1224 (0.07)	<0.001
Coronary artery disease	4465 (0.06)	2117 (0.12)	<0.001
Cerebrovascular accident	4104 (0.06)	1664 (0.09)	<0.001
Chronic Kidney disease	586 (0.01)	687 (0.04)	<0.001
Cancer	2368 (0.03)	1977 (0.11)	<0.001
Chronic Obstructive Pulmonary Disease	2359 (0.03)	1453 (0.08)	<0.001
Sleep apnea	66 (0.001)	41 (0.002)	<0.001
Rheumatoid arthritis	144 (0.002)	190 (0.01)	<0.001
Atrial fibrillation	916 (0.01)	537 (0.03)	<0.001
Chronic liver disease	553 (0.01)	419 (0.02)	0.75
Pregnancy	13 (0.0002)	2 (0.0001)	<0.001
Lower leg fracture or surgery	2935 (0.04)	1121 (0.06)	<0.001
Mortality	5560 (7.78)	4290 (24.0)	<0.001

Follow-up time: non-NTS group: 4.94 (3.93) years; NTS group: 6.30 (3.67) years. The chi-square test was used to examine categorical data. * t-Test examined continuous data.

**Table 2:** Incidence and hazard ratios for deep vein thrombosis (DVT) and pulmonary embolism (PE), comparing patients with and without NTS.

Variables	Event			Crude HR ^‡^		Adjusted HR ^§^		SHR	
	*n*	PY	Rate ^†^	(95% CI)		(95% CI)		(95% CI)	
***DVT***									
Non-NTS	215	450,270	4.77	1.00	-	1.00	-	1.00	-
NTS	109	88,183	12.36	2.54	(2.02 to 3.20) ***	2.35	(1.86 to 2.98) ***	1.83	(1.44 to 2.31) ***
Gender									
Female	154	248,049	6.21	1.00	-				
Male	170	290,404	5.85	0.94	(0.75 to 1.17)				
Age									
18–39	22	164,576	1.34	1.00	-	1.00	-	1.00	-
40–64	82	209,177	3.92	2.91	(1.82 to 4.66) ***	2.49	(1.55 to 4.01) ***	2.93	(1.47 to 3.90) ***
≥65	220	164,700	13.36	9.68	(6.24 to 15.0) ***	6.41	(4.04 to 10.2) ***	5.58	(3.38 to 9.22) ***
Comorbidities									
No	142	432,486	3.28	1.00	-	1.00	-	1.00	-
Yes	182	105,967	17.18	5.06	(4.05 to 6.32) ***	2.58	(2.02 to 3.29) ***	2.09	(1.60 to 2.73) ***
***PE***									
Non-NTS	109	450,270	2.42	1.00	-	1.00	-	1.00	-
NTS	53	88,183	6.01	2.46	(1.77 to 3.41) ***	2.36	(1.69 to 3.30) ***	1.84	(1.30 to 2.60) ***
Gender									
Female	76	248,049	3.06	1.00	-				
Male	86	290,404	2.96	0.96	(0.71 to 1.31)				
Age									
18–39	15	164,576	0.91	1.00	-	1.00	-	1.00	-
40–64	43	209,177	2.06	2.28	(1.26 to 4.10) ***	2.05	(1.13 to 3.71) *	1.96	(1.08 to 3.56) *
≥65	104	164,700	6.31	7.09	(4.12 to 12.2) ***	5.27	(2.97 to 9.37) ***	4.51	(2.46 to 8.26) ***
Comorbidities									
No	82	432,486	1.9	1.00	-	1.00	-	1.00	-
Yes	80	105,967	7.55	4.09	(2.99 to 5.59) ***	2.15	(1.52 to 3.03) ***	1.68	(1.15 to 2.47) **

* *p* < 0.05, ** *p* < 0.01, *** *p* < 0.001. ^†^ Rate, incidence rate, per 10 000 person-years. ^‡^ Crude HR: relative HR. ^§^ Adjusted HR: adjusted HR controlling for exposure, age, and comorbidities. SHR: competing risk of death; Comorbidity: patients with hypertension, diabetes, hyperlipidaemia, coronary artery disease, cerebrovascular accident, chronic kidney disease, cancer, chronic obstructive pulmonary disease, sleep apnea, rheumatoid arthritis, atrial fibrillation, chronic liver disease, pregnancy, lower leg fracture, or surgery were classified as the comorbidity group. DVT: deep vein thrombosis; PE: pulmonary embolism; PY: person-years. PSM: propensity score matching.

**Table 3:** Incidence and hazard ratios for DVT and PE among patients stratified by sex, age, and co-morbidities.

Variables	Salmonellosis						Compared to Control				*p* for Interaction
	Yes			No			Crude HR ^‡^		Adjusted HR ^§^		
	Events			Events							
	*n*	PY	Rate ^†^	*n*	PY	Rate ^†^	(95% CI)		(95% CI)		
***DVT***											
Gender											0.86
Female	54	42,315	12.8	100	205,734	4.86	2.61	(1.87 to 3.63) ***	2.34	(1.67 to 3.28) ***	
Male	55	45,868	12.0	115	244,536	4.70	2.48	(1.80 to 3.42) ***	2.26	(1.63 to 3.14) ***	
Age, years											<0.001
18–39	16	30,710	5.21	6	133,866	0.45	11.4	(4.44 to 29.0) ***	5.95	(2.22 to 15.9) ***	
40–64	38	34,158	11.1	44	175,018	2.51	4.37	(2.83 to 6.74) ***	3.40	(2.16 to 5.34) ***	
≥65	55	23,315	23.6	165	141,385	11.67	1.99	(1.47 to 2.70) ***	1.68	(1.23 to 2.30) **	
Comorbidity											0.54
NO	33	61,849	5.34	109	370,637	2.94	1.82	(1.24 to 2.69) ***	2.33	(1.58 to 3.45) ***	
YES	76	26,334	28.9	106	79,633	13.3	2.10	(1.57 to 2.83) ***	2.17	(1.61 to 2.93) ***	
Follow-up time, months											0.06
≤2	20	103	1950	2	26	775	0.70	(0.31 to 1.58)	0.71	(0.31 to 1.67)	
3–12	25	868	288	10	944	106	1.25	(0.72 to 2.19)	1.42	(0.79 to 2.56)	
>12	64	87,212	7.34	97	449,300	2.16	1.84	(1.38 to 2.44) ***	1.75	(1.31 to 2.34) ***	
***PE***											
Gender											0.06
Female	20	42,315	4.73	56	205,734	2.72	1.73	(1.04 to 2.89) **	1.65	(0.98 to 2.77)	
Male	33	45,868	7.19	53	244,536	2.17	3.26	(2.11 to 5.04) ***	3.04	(1.94 to 4.75) ***	
Age, years											0.004
18–39	10	30,710	3.26	5	133,866	0.37	8.48	(2.90 to 24.8) ***	6.72	(2.23 to 20.3) ***	
40–64	20	34,158	5.86	23	175,018	1.31	4.39	(2.41 to 8.00) ***	3.28	(1.75 to 6.13) ***	
≥65	23	23,315	9.86	81	141,385	5.73	1.77	(1.11 to 2.82 ) **	1.54	(0.96 to 2.47)	
Comorbidity											<0.001
NO	24	61,849	3.88	58	370,637	1.56	2.47	(1.54 to 3.98) ***	3.05	(1.89 to 4.92) ***	
YES	29	26,334	11.01	51	79,633	6.40	1.70	(1.08 to 2.68) **	1.74	(1.10 to 2.77) *	
Follow-up time, months											0.73
≤2	2	103	195	8	26	3100	1.16	(0.25 to 5.38)	1.12	(0.22 to 5.73)	
3–12	10	868	115	15	944	159	1.94	(0.87 to 4.33)	2.41	(1.04 to 5.55) *	
>12	97	87,212	11.1	29	449,300	0.6	1.57	(1.04 to 2.37) *	1.56	(1.03 to 2.38) *	

* *p* < 0.05, ** *p* < 0.01, *** *p* < 0.001. ^†^ Rate, incidence rate, per 10 000 person-years. ^‡^ Crude HR: relative HR. ^§^ Adjusted HR: adjusted HR controlling for age, sex, and comorbidities. Comorbidity: patients with hypertension, diabetes, hyperlipidaemia, coronary artery disease, cerebrovascular accident, chronic Kidney disease, cancer, Chronic Obstructive Pulmonary Disease, sleep apnea, rheumatoid arthritis, atrial fibrillation, chronic liver disease, pregnancy, lower leg fracture, or surgery were classified as the comorbidity group. DVT: deep vein thrombosis; PE: pulmonary embolism; PY: person-years.

**Table 4:** Cox proportional hazard regression models for risk of DVT and PE in primary and sensitivity analyses.

Variable	Event			Crude HR ^‡^		Adjusted HR ^§^		SHR	
	*n*	PY	Rate^†^	(95% CI)		(95% CI)		(95% CI)	
**Primary analysis (frequency matching)**									
***DVT***									
non-NTS	215	450,270	4.77	1.00	-	1.00	-	1.00	-
NTS	109	88,183	12.36	2.54	(2.02 to 3.20) ***	2.35	(1.86 to 2.98) ***	1.83	(1.44 to 2.31) ***
***PE***									
non-NTS	109	450,270	2.42	1.00	-	1.00	-	1.00	-
NTS	53	88,183	6.01	2.46	(1.77 to 3.41) ***	2.36	(1.69 to 3.30) ***	1.84	(1.30 to 2.60) ***
**Sensitivity analysis (PSM)**									
***DVT***									
non-NTS	256	425,265	6.02	1.00	-	1.00	-	1.00	-
NTS	102	88,109	11.6	1.90	(1.51 to 2.38) ***	2.17	(1.72 to 2.73) ***	1.69	(1.34 to 2.13) ***
***PE***									
non-NTS	113	425,265	2.66	1.00	-	1.00	-	1.00	-
NTS	47	88,109	5.33	1.98	(1.41 to 2.78) ***	2.16	(1.53 to 3.05) ***	1.71	(1.21 to 2.40) **

** *p* < 0.01, *** *p* < 0.001. ^†^ Rate, incidence rate, per 10,000 person-years. ^‡^ Crude HR: relative HR. ^§^ Adjusted HR: adjusted HR controlling for exposure, age and comorbidities. SHR: competing risk of death. DVT: deep vein thrombosis; PE: pulmonary embolism; PY: person-years. PSM: propensity score matching.

## Supplementary Materials

The following are available online at https://www.mdpi.com/1660-4601/17/10/3567/s1, Table S1: Comparison of demographics and comorbidity between individuals with and without nontyphoidal salmonellosis in propensity score matched cohort.
