# Supplementary material for: The Risk of Subsequent Deep Vein Thrombosis and Pulmonary Embolism in Patients with Nontyphoidal Salmonellosis: A Nationwide Cohort Study
Source: Int J Environ Res Public Health. 2020 May 19;17(10):3567. doi: 10.3390/ijerph17103567 (PMC7277939; doi:10.3390/ijerph17103567)
Supplement: Supplementary file 1 [file ijerph-17-03567-s001.pdf]

Table S1. Comparison of demographics and comorbidity between individuals with and without nontyphoidal salmonellosis in propensity score matched cohort.

Propensity Score Matched			
	**Nontyphoidal Salmonellosis**		
	**No (N = 71368)** *n* **(%)**	**Yes (N = 17842)** *n* **(%)**	**SMD**
Age, years			
18-39	4718 (26.4)	21,306 (29.9)	0.08
40-64	6509 (36.5)	27,354 (38.3)	0.04
≥65	6615 (37.1)	22,708 (31.8)	0.11
Mean (SD) *	55.4 (19.7)	53.3 (19.2)	0.11
Gender			0.07
Female	8044 (45.1)	29,677 (41.6)	
Male	9798 (54.9)	41,691 (58.4)	
Comorbidities	7949 (44.6)	34,984 (49.0)	0.09
Hypertension	4312 (24.2)	17,290 (24.2)	0.00
Diabetes	2853 (16.0)	11,418 (16.0)	<0.001
Hyperlipidemia	1224 (6.9)	7668 (10.7)	0.14
Coronary artery disease	2117 (11.9)	8460 (11.9)	<0.001
Cerebrovascular accident	1663 (9.3)	6564 (9.2)	0.004
Chronic Kidney disease	687 (3.9)	4421 (6.2)	0.11
Cancer	1977 (11.1)	7897 (11.1)	<0.001
Chronic Obstructive Pulmonary Disease	1452 (8.1)	4000 (5.6)	0.10
Sleep apnea	41 (0.2)	2862 (4.0)	0.27
Rheumatoid arthritis	190 (1.1)	1588 (2.2)	0.09
Atrial fibrillation	537 (3.0)	2279 (3.2)	0.01
Chronic liver disease	419 (2.3)	1807 (2.5)	0.01
Pregnancy	2 (0.01)	12 (0.02)	0.01
Lower leg fracture or surgery	1121(6.3)	4850 (6.8)	0.02
Mortality	8245(11.6)	4291 (24.1)	0.33

Follow-up time: NTS group: 4.94 (3.93) years; non-NTS group: 5.95 (3.75) years. Chi-square Test examined categorical data. * t-Test examined continuous data. SMD: *Standardized Mean Difference* (less than 0.1 means no differences).
